# Correction: Barriers to care for dependent older adults: Brazilian Primary Health Care managers’ perspective

**DOI:** 10.1371/journal.pone.0316657

**Published:** 2024-12-26

**Authors:** Jonas Loiola Gonçalves, Raimunda Magalhães da Silva, Girliani Silva de Sousa, Indara Cavalcante Bezerra, Christina César Praça Brasil, Luiza Jane Eyre de Souza Vieira, Fernanda Colares de Borba Netto, José Maria Ximenes Guimarães, Maria Cecilia de Souza Minayo

The affiliation for the fifth author is incorrect. Christina César Praça Brasil is not affiliated with #1 but with #2: Graduate Program in Collective Health, University of Fortaleza, Fortaleza, Ceará, Brazil.
