# Supplementary material for: Barriers to care for dependent older adults: Brazilian Primary Health Care managers’ perspective
Source: PLoS One. 2024 Oct 9;19(10):e0309309. doi: 10.1371/journal.pone.0309309 (PMC11463773; doi:10.1371/journal.pone.0309309)
Supplement: S1 File — (DOCX) [file pone.0309309.s001.docx]

**Summary and cross-sectional analysis of all the respondents**

Once data organization is standardized, carry out a cross-sectional synthesis of the interviews performed, drawing attention to the most relevant points. It is a consolidated statement of all the interviews conducted by you, considering all participants, e.g. all interviews with the older adults, all interviews with family caregivers, and so on, following the **roadmap for organizing and systematizing the data** described above. Aspects not mentioned in the data organization and systematization roadmap may appear in the synthesis and cross-sectional analysis, and they should be added in this item.

**MANAGER**

**Initial data**

Name:

Age:

Place of birth:

Ethnicity/skin color:

Residing in the municipality/neighborhood of:

Seniority in the service:

Professional education:

Seniority in the position/title:

**Perception of the manager about the dependence of older adults and initiatives to support the older adult and his/her family**

- Manager’s understanding of social, physical, mental, and cognitive dependence from the viewpoint of health care and social assistance.
- Initiatives that exist and other that could be implemented to support these families.

**Suggested Analysis Categories**

**MANAGERS**

1.Initial Data – Name, age, gender, seniority in the position, etc.

2.Perception of the manager about the dependent older adult

3.Initiatives to improve the care for dependent older adults

**Available at:** [Research Manual Situational Study of Dependent Older Adults Living with Their Families - Ciência & Saúde Coletiva (scielo.org)](https://data.scielo.org/dataset.xhtml?persistentId=doi:10.48331/scielodata.8NEDAK)

**Results**

| **Thematic** | **Results** | **Interpretation** |
| --- | --- | --- |
| **Barriers caused by problems in family care for the dependent older adults** | You can have ten children who will pass the responsibility on to another, and no one will want to take care of them. We are often forced to call the social service to pressure the family to provide care, saying it is an obligation. If you don’t have a bath, a clean house, affection, and a distraction, this impacts society and management and will demand a little effort from everyone. (Manager 1 – Araranguá)  We understand that all the problems are at the family level. (Manager 1 – Manaus)  Most cases that arise for social assistance are family negligence. (Manager 2 – Belo Horizonte) | In general, the managers pointed out that several factors exceed the institutional possibilities as they exist today for consolidating care for dependent older adults: |
|  | Their children grew up. They left, and the older adults lived alone without any care. So, our difficulty is providing reasonable assistance to this older adult without him having a caregiver (Manager 1 – Rio de Janeiro/RJ).  There is great neglect by family members. It may be due to a lack of guidance, information, or something else. I know some situations could be prevented (Manager 2 – Manaus).  There is no family. She cannot move around and stays home. We have some older adults in the Home Care Service. She doesn’t get better because the family doesn’t let her date or go dancing. She needs a partner. Her loneliness is not about family but emotional (Manager 2 – Araranguá). | Managers struggle to plan care provision because the professionals’ prescriptions need to be followed by someone who can understand and maintain them |
|  | The family member is unprepared to take care of this older adult. We realize the great difficulty of the family. They get here sometimes needing help. (Manager 4 – Araranguá)  The weakest side is that of the family because it is the one that has no resources. It’s what is criminalized when missing. You criminalize the family that neglects, takes care of badly, or mistreats, but you don’t verify under what circumstances that family is being forced to take care of. The State is sovereign there, just watching. (Manager 2 – Belo Horizonte)  Families are often unable to take the older adult to the health unit... which makes it very difficult. (Manager 1 – Fortaleza)  There needs to be more proper nutrition. Perhaps the family needs to prepare. (Manager 3 – Araranguá) | Participants point out that the family’s unpreparedness to address the specific situations of older adults directly affects care management. However, they add that there needs to be more financial resources and State support for older adults. This omission leads to fragmenting, disrupting, and upkeeping good practices. |
|  | “Family members say they will help, but they only take ownership of the retirement benefit this older adult receives, but they do not provide care!” (Manager 3 – Belo Horizonte)  They think that at home, they are useless older adults. It is often because they have a grandson at home or a family member who does not have the conversation they wanted to have. (Manager 1 – Manaus)  Many of these seniors may need a family or a robust social circle. They need the government to be concerned and care for them, especially medication time, food, and bathing because he is there alone. (Manager 2 – Belo Horizonte) | The statements mentioned above reveal different nuances: the most general is that managers complain about the negligence and lack of preparation of family members. However, one of them highlights the difficulties of the poorest people in providing care, adequate food, and medication for older adults. They should not be charged with negligence. Another of them adds: |
|  | If the caregiver were better prepared, he would sometimes succeed. However, he needs to be more prepared, untargeted, and a little left out. He is also tired and repeats: I can’t. (Manager 3 – Araranguá)  Older adults do not just need to be fed and bathed but also receive attention and affection. Many family members who are caregivers do not have patience as they are sick, too, because it is complicated. (Manager 2 – Manaus). | Today, we observe a paradox experienced by families with older adults in their homes. Soon these are usually ageism victims. From the non-recognition of older adults as essential people in the family circle or by society, the burden and lack of preparation of those caring for them and their impoverishment emerge, along with the lack of financial conditions to pay for formal caregivers. |
| ***Lack of priority in Primary Care programming*** | Public health cannot reach all older adults. These patients keep repeating their comings and goings in PHC, repeating... because public health does not have the means for everything. (Manager 2 – Araranguá)  This Greater Care program offers caregivers for elderly people who are in areas of the Social Assistance Reference Center (CRAS), are in areas of high social vulnerability. However, it is a cut-out within the cut-out. (Manager 4 – Belo Horizonte). | Managers point out that care for dependent older adults is a huge challenge for the Brazilian health system, as the SUS still “*does not have the means for everything*”. Therefore, the demands regarding aging emerge as an issue. |
|  | Regarding scope and reception, care for the elderly still leaves much to be desired”. (Manager 2 – Belo Horizonte)  It’s a big problem, and it will increase a lot. I want to see later the problem with home admission, the bedridden person, the weaknesses of social relationships, and the massive demand on the Public Prosecutor’s Office. (Manager 1 – Teresina)The current Family Health strategy can only meet some of the demands. It is not just primary care but specialized care as well. (Manager 3 – Araranguá) | Managers point out that the insufficient Health Care Network is an obstacle, particularly in care and home care. Participants understand that this need is more acute in failures in the reception and follow-up due to the following: |
|  | The challenges are to serve this population, which is growing in the city and demands shelter, care, and protection. (Manager 4 – Belo Horizonte)  We have a massive demand from older adults. This demand is huge here in Teresina. (Manager 1 – Teresina)  Our units are overloaded. We have several inhabitants well above what the PNAB recommends for each strategy. So, this hinders providing more detailed care for older adults. (Manager 4 – Araranguá)  We have in the city of Belo Horizonte, the Greater Care program, which is social assistance and that it is having a result, but it needs to be expanded, we see more and more the need for greater care of the population and these caregivers in the low-income population, which really has no one to take care of (Manager 1 – Belo Horizonte). | Managers point out that older adults’ demands are far more significant and repeated than the few professionals to assist them in the setting of Primary Care’s responsibility, which hinders universal care and social protection; that is, they are implicitly or explicitly seen and addressed as a problem. |
|  | The team already supports other things. So, on this health issue, the PAIF team is very overloaded. This situation brings uncertainty about how to proceed with this professional. (Manager 3 – Belo Horizonte)  This is one of the most significant difficulties for municipalities today. Strategies for work bottlenecks and professional overload. (Manager 3 – Araranguá)  The teams try their best, but we need more support. We have partner institutions, but more is needed. Our population covered by the Family Health strategy is an extremely old population. (Manager 1 – Rio de Janeiro). | In their statements, managers generally point out that professionals try their best, given the barriers they face, with the help of third-sector institutions. However, they need to meet the needs adequately. |
|  | There was a time when I had nothing else to do regarding the professionals. The secretariat cannot absorb all the patients for therapy with the psychologist. Social assistance is unable to visit everyone. (Manager 1 – Araranguá)  There is no way for primary care to achieve this. Visits every fortnight. We have primary care, and we advise. So, what happens? They call me a lot, and I call the nurse: ‘Look, can you go there, give that person some attention?’ So, we adapt to the incoming demand. (Manager 4 – Araranguá) | Concretely, this situation results in the frailest older adults not receiving the necessary care and treatment continuity. Managers showed they were aware of the problems. Several of them are very sensitive to the suffering of older adults and seek to improve the quality of services, usually without great success |
|  | Professionals only try to put out the fire and do what is agreed. So, we go there and do it and say we did it. (Manager 3 – Araranguá)  We are concerned with immediate action. Professionals are suffocated in resolving problems. They want to know about something other than monitoring. They want to solve the public’s problems. They need to think about quality. (Manager 1 – Teresina) | The work processes to provide greater resolution are seen by several managers as an attempt to put out the fire as they focus on solving specific situations with questionable quality: |
| ***Failures in recruiting and training professionals and lack of a specific public policy*** | We work with a team of professionals who need to gain the minimum skills to provide this care to the public. (Manager 1 – Fortaleza) | Managers point out that developing skills and competencies for caring for older adults’ health needs to be more specific, specific, and minimal. Educational failures reverberate in Primary Care and Home Care services. |
|  | The change of professionals, nurses, and doctors with their profiles and role needs more articulation. (Manager 4 – Araranguá)  When I arrived here, we had a social worker at the unit. Then, we started losing specialists. There was a social worker who helped us a lot. Many servants got scared and asked to leave with the expanded Family Health. (Manager 1 – Rio de Janeiro)  In the daily life of the manager who works in health, there need to be more resources for older adults, and the main thing needed is human resources. (Manager 2 – Fortaleza)  The “Mais Médicos” (More Doctors) contract for doctors is over. We only had one doctor in the unit. So, it is challenging. The professionals recruited are per service provided. They come and go, both doctors and nurses. Employment ties are fragile. (Manager 1 – Fortaleza). | Along with the complex care process, turnover in health services has become a widespread problem in the Brazilian health sector after the rules for outsourcing Primary Care services. These precarious work ties and the shortage of personnel reduced the care capacity. There is no guarantee of continuity, and the flow of the treatment process and access to adequate care has been broken. |
|  | There are no goals to be achieved for older adults, as in the case of children. I have yet to set a goal to say how many appointments an older adult should have. (Manager 1 – Fortaleza)  The service needs to plan care. This situation needs to be reversed because we are still very reactive and waiting for the incoming demand. (Manager 2 – Belo Horizonte) | The lack of a priority agenda and planning focused on the health of older adults in Primary Care and Home Care is seen by managers as a negligence factor in the daily practice of services. |
|  | We left college, doctors and nurses, with our minds on prenatal care, childcare, TB, leprosy, immunization, hypertension, and diabetes care, so we fragmented ourselves. (Manager 1 – Rio de Janeiro)  We received interns from several undergraduate courses. Only some bring initiatives geared toward older adults. Most are for women and children. The municipality itself conforms to this. (Manager 1 – Fortaleza).  So, you know, sometimes, there is a limit to the program. As long as he has the family and the services there, the family, the program is maintained, but an elderly person who does not have a family caregiver, a rearguard, then it is impossible for us to maintain the program Greater Care (Manager 2 – Belo Horizonte). | Faced with several barriers to these two essential services, managers consider that the problem begins in Brazilian public and private universities, where there needs to be more interest in training to take care of older adults’ health. |
|  | What I demand from senior managers are policies aimed at older adults. Most target children, women of childbearing age, and little is said about older adults. (Manager 1 – Brasília)  Most dependent older adults result from poor monitoring of hypertension and diabetes. In other words, the health system does not have an adequate care model for monitoring chronic conditions. (Manager 2 – Fortaleza)  In relation to care, the big issue is often politics today within the city of Belo Horizonte, because we see the needs becoming more and more present (Manager 1 – Belo Horizonte). | Not only training is questioned by managers, but mainly the lack of a public policy to strengthen elderly care. |
