# Supplementary material for: Barriers to care for dependent older adults: Brazilian Primary Health Care managers’ perspective
Source: PLoS One. 2024 Oct 9;19(10):e0309309. doi: 10.1371/journal.pone.0309309 (PMC11463773; doi:10.1371/journal.pone.0309309)
Supplement: Attachment — Submitted filename: Response to reviewers.docx [file pone.0309309.s002.docx]

**Response to reviewers**

**Introduction**
1. The Brazilian Institute of Geography and Statistics (IBGE) published the 2022 Demographic Census. I suggest updating the demographic data presented in the article.
Response: Population aging is almost widespread globally, albeit in heterogeneous epidemiological rhythms. The 2023 Brazilian Census reveals that the elderly population has reached 31,2 million, representing 14,7% of the brazilian, the growth of this population represented 39.8% from 2012 to 2021¹.

2. The results presented are part of a larger research, as the authors mention (lines 126-131). However, the choice of the objective of this article with other aspects found is not sufficiently clarified. It inappropriate to say “the entire collection of this knowledge is registered in a thematic issue of the Ciência & Saúde Coletiva jornal” (lines 160-161). There are in this publication, for example, other articles that do not deal with the same theme.
Response: We present a preview of the results in a collection registered in a thematic issue of the Ciência & Saúde Coletiva journal^23^. The managers, the main stakeholders in this study were heard through a semi-structured interview at a pre-established date, time, and place.

3. Programa Acompanhante de Idosos (PAI) of the Municipal Secretary of Health of São Paulo is the correct name. It’s necessary to change (line 82) “São Paulo has the Acompanhamento ao Idoso (“Elderly Monitoring”)...”.

**Response:** São Paulo has the “Programa Acompanhamente de Idosos” (PAI) (“Elderly Monitoring”) Program, which provides a multidisciplinary team to support the most fragile and dependent, including meeting their needs of daily life.

**Methods**

1. Presents theoretical foundation, description of procedures and ethical aspects of the research. However, It’s appropriate to rephrase the paragraph (lines 138-143) “... all of them (including Araranguá) share that they are hubs for gathering the demand of SUS users since they have all the equipment and resources for primary, secondary, tertiary, and ultra-specialized care” The reference (22) is insufficient to characterize the health care network. Publications in reference (23) have more adequate paragraphs aboutit.

Response: However, all of them (including Araranguá) are hub regions for meeting the demands of the Unified Health System (SUS), providing users with the equipment and resources necessary for care in the areas of primary, secondary, tertiary, and ultra-specialized care^22-23^.

2. 16 study participants in the 8 cities. It’s necessary to describe this distribution, as also the responsibilities inherent in managing care for dependent older adults.
Response: Study participants form a sample of 16 managers from five different country regions, distribution in: Araranguá – SC (4) , Brasília – DF (1), Fortaleza-CE (2), Manaus – AM (2), Porto Alegre – RS (1), Belo Horizonte – MG (4), Rio de Janeiro - RJ (1) e Teresina – PI (1). They are also selected by convenience and because they are firmly integrated with the local administration and with responsibilities inherent in managing care for dependent older adults, managers who coordinate and are responsible for managing basic care units, programs and specialized services offering health care to dependent elderly people.

3. It’s advisable to specify the software used in the research (line 172).

Response: The statements were fully transcribed and organized manually, without software support.

**III. Results**

It’s appropriate to clarify acronyms and citation some excerpts from the interviews – “PAIF”, “Mais Médicos”, “PNAB”. Note: also in other parts of the article, such as “PHC”, “SUS”.

Response: São Paulo has the “Programa Acompanhamente de Idosos” (PAI) (“Elderly Monitoring”)

In Rio de Janeiro, primary care offers care to bedridden older adults. However, the health workers and the Primary Health Care (PHC) ’ team recognize they can do very little and cannot attend to all older adults in need^6^.

Structuring an Elderly Care Network regulated by Primary Care requires close communication between the Unified Health System (SUS) and the Unified Social Assistance System (SUAS).

The team already supports other things. So, on this health issue, the Family Protection and Comprehensive Care Service (PAIF) team is very overloaded. This situation brings uncertainty about how to proceed with this professional. (Manager 3 – Belo Horizonte)

Our units are overloaded. We have several inhabitants well above what the National basic care policy (PNAB) recommends for each strategy. So, this hinders providing more detailed care for older adults. (Manager 4 – Araranguá)

The “Programa Mais Médicos” (Program More Doctors)

**IV. Discussion**

The dimension “barriers caused by problems in family care for the dependent older adults” has not been sufficiently analysed.

Response:

Family barriers that permeate the accelerated growth of the older population contradict the low birth rates, as well as changes in marriage rates and the insertion of women in the job market. It is evident that Brazilian women are increasingly assuming the role of provider, but still maintain responsibility for the care of those who are dependent, changes that directly affect the ability of families to guarantee and offer care to the elderly population^27^.

Furthermore, the work of these family caregivers is often uninterrupted and solitary, without the support of health services and public protection policies. There is a need for public support policies for dependent elderly people and their caregivers, with the aim of overcoming barriers and subsidizing better care for everyone^27-28^.

There are participants who have been worked in Belo Horizonte. However They didn’t talk about ”Greater Care”. Fact that deserves discussion.
Response: We complement the interpretation, of this request.

The argument in lines 386-394 doesn’t establish a dialogue necessary with lack of knowledge of the subjects of the policy.
The Policy document^17^ mentions dependent older adults in two stages: in the introduction and the chapter addressing the integrality of the actions, highlighting a concern when managers are unaware of the policy issues, especially regarding the themes of dependent elderly people. Because the oldest with functional difficulties stood at 2%-4% at the time of writing. That may be why the emphasis was on healthy aging, and the loss of autonomy deserved only general considerations. In the 2013 National Health Survey, they found 8.4% of older adults dependent on others to perform the activities of daily living, and 22.0% for instrumental activities, showing that the percentage of older adults who are losing autonomy increases while the number of those aged 80 and over is growing rapidly^30^.

It’s necessary discuss more appropriately with the results the argument “It is a consensus among the authors that a health hospital-and-medical professional-centered system alone will have to be modified” (lines 450-451).

Response:

It is a consensus among the authors that a health system centered on the hospital and the medical professional is not sufficient to meet the demands of dependent elderly people, there is a need for training, public policies, and dialogue with the whole of society. Investments in Primary Care to reduce and consolidate care are necessary in an elderly society whose future is already present, including in low- and middle-income countries. The authors highlight that Japan has the most consolidated primary care services. The Japanese government implemented the “Community-Based Integrated Care System” to offer practical support to people at the end of life, guided by four elements: self-help, mutual help, social solidarity care and government care^36^.
