# Supplementary material for: Barriers to care for dependent older adults: Brazilian Primary Health Care managers’ perspective
Source: PLoS One. 2024 Oct 9;19(10):e0309309. doi: 10.1371/journal.pone.0309309 (PMC11463773; doi:10.1371/journal.pone.0309309)
Supplement: Attachment — Submitted filename: Response to reviewers.docx [file pone.0309309.s003.docx]

**Response to reviewers**

First, we would like to thank the reviewers for their efforts with the manuscript. We seek to promptly respond to all the required suggestions. In this way, fostering the presentation of the study to the Brazilian reality and the international community, considering that the theme both in national and international territory needs even more a rapid debate between the various countries. In this sense, the material based on the suggestions and publication in this important international journal is seen as an important link for the dissemination based on good practices in science and to contribute to the debate of society, especially managers and formulators of public policies.

**REVIEWER 1**

**Introduction:**

1- It is bureaucratic and, at times, generic. It needs to be very clear about what is meant by "dependent adult". It needs to explain to the foreign reader in a synthetic way how PHC is organized in Brazil and how it is articulated in the elderly care network, etc. Make it clearer what it is, including SUS!!!

R. We seek to meet this suggestion and leave the text as follows: It is important to note that in the Brazilian situation, before the promulgation of the Federal Constitution, the elderly population suffered from incipient and charitable government actions. From its institution, the SUS was created, a health instance focused on the universal right to health. An articulation with the Unified Social Assistance System (SUAS) emerged, in the search to overcome difficulties and guarantee social protection for the population, based on support for people, families and their community contexts.

2- References are missing in paragraphs 4, 6, 7, 8, 9 and many others.
misconception:

R. Reviewed and presented; we apologize for this misconception presented in review 1.

3- “In Brazil, PHC’s attributions are promoting health, preventing diseases, and providing care7”. In this sentence, you need to be careful, since it cannot be just in Brazil, as they are attributes of (non-selective) APS internationally.

R. Based on the suggestion, we adapted it to the following text: In the case Brazil and other international contexts, the attributions of PHC’s (non-seletive) are promoting health, preventing diseases, and providing care.

4- In the sentence “Structuring an Elderly Care Network regulated by Primary Care requires close communication between the Unified Health System (SUS) and the Unified Social Assistance System (SUAS), as recommended in other countries”, a reference is needed at the end of the sentence and, Also, justify why? It was confusing and also does not make it clear to the non-Brazilian reader what SUS and SUAS mean.

We seek to meet this suggestion and leave the text as follows: It is important to note that in the Brazilian situation, before the promulgation of the Federal Constitution, the elderly population suffered from incipient and charitable government actions. From its institution, the SUS was created, a health instance focused on the universal right to health. An articulation with the Unified Social Assistance System (SUAS) emerged, in the search to overcome difficulties and guarantee social protection for the population, based on support for people, families and their community contexts.

5- In general, the introduction paragraphs are not articulated and are only descriptive, lacking some criticality in the writing and approach. The problem is not well discussed in the introduction. According to the research question, discuss PHC and its relationship with elderly care in Brazil and around the world. The question is not thematized in the introduction, which raises relevant questions, although disjointed and without debate appropriate to the problem presented. The barriers were not discussed in the text or were poorly explained in the description.

R. Based on the reordering and adjustments suggested above, we sought to have taken into account the suggestions of reviewer 1, taking into account this comment.

**Materials and methods**:

6- There are many references missing in the paragraphs to strengthen the argument.

R. Reviewed and presented; we apologize for this misconception presented in review 1.

7- Where and how was it used in hermeneutic-dialectic analysis? There is nothing to demonstrate its use. In what aspects did HD help in understanding the results and their discussion?

R: We complement the material with the following paragraphs, seeking to meet the present suggestion:

We developed a qualitative study anchored in the hermeneutic theoretical framework that is based on the understanding of social processes, based on the communication of daily life, common sense, considering that the human being is permeated by its historicity, and the interconnections come from social and cultural contexts20-21.

Based on the experiences, we tried to analyze a consensus of the experiences, senses, meanings and symbols contained in the participants' testimonies. Thus, a comprehensive reflection emerges on the barriers pointed out by PHC health managers regarding the adequate provision of health care to dependent elderly people living at home20-21.

8- Is HD possible without data triangulation? Why does this research, the result of a larger and richer research as a whole, boil down to treating the perception of managers without triangulating it with other data, including sociodemographic data?

R. We introduce and justify the triagulation as presented below: The study is guided by the assumptions of triangulation, considering of the participation of places, people, researchers, theories, and the intra-method20. Involving the understanding of the work of PHC (health workers, professionals who work in the units and managers) in the face of the interlocutions of the situation of the elderly, their families and family caregivers6.

9- Why and how were the cities chosen? Why is one of them not a state capital and what is the implication of this in the analyses? 10- Why 4 managers in Araranguá and fewer in the others?

R. View limitations at study inserted conform review 2.

11- I suggest a map with the location of these municipalities in the country for better visualization.

R. Map presented from the inclusion of figure 1, as requested.

12- Also, a table is needed with data from these municipalities: population, PHC coverage, other services, number of elderly people, epidemiological data, etc.
R. We seek to meet this suggestion without losing the essence of qualitative research, since its currents are not based on positivist assumptions.

13- The authors state that the choice was made for convenience, but what is appropriate in qualitative research is an intentional choice!!! (could you justify it better?).

R. We follow the suggestion and present the following text: Selected by intentionality and because they are firmly integrated with the local administration and with responsibilities inherent in managing care for dependent older adults, managers who coordinate and are responsible for managing basic care units, programs and specialized services offering health care to dependent elderly people.

14- Regarding the interviews, you need to provide details with COREQ and not just indicate what you used, as important details are missing (Who carried them out? See Quoreq and try to bring up aspects that clarify the conditions of data production). The average interview time was 30 minutes? (Only? What depth is possible in such short interviews?). Explain in the method how you arrived at the categories/topics.

R. This suggestion was heeded and consolidated throughout the manuscript.

**Results**

15- The fragments are interesting and rich. However, there are two general problems: the description of the results is VERY synthetic and does not explore the wealth of information. In this sense, the description is limited to repeating the managers' arguments as if the statements contained a truth in themselves; 16- As a result, the text contains many fragments (empirical results), but does not explore them and, consequently, leaves it up to the reader to interpret the results. In my understanding, this choice impoverished many of the data found and compromised the entire article, making it fragile and without sufficient argumentative strength. 17- There would be a need to rewrite all the results, even if the chosen topics were maintained, but described with more attention and more criticality (the description does NOT mean repetition of arguments, but elaboration of text that synthesizes the ideas contained explicitly or implicitly in the managers' fragments). 18- Are the categories/topics common for all elected municipalities? Is there no diversity among managers? Wouldn't this be an aspect worth considering?

R. Based on the suggestions of both reviewers, we seek to respond promptly to this request, and we hope that our efforts have been able to achieve this request.

**Discussion**
19- I feel that the discussion does not fulfill the promise of hermeneutic-dialectic analysis. Or I would need to demonstrate the strength of dialectics in writing. The text is quite descriptive and, to some extent, repeats the results without sufficient criticality, historicity or dialectics.

R. We have adopted the hermenutic path based on the suggestions in the methods, we have revisited all the material, especially on this topic, so we hope to have responded to this suggestion.

20- In the way they judge families, there would be a need to consider the different family contexts across the country (demographic and socioeconomic data added to inferences that consider national complexity in different regions of the country).

R. We seek to bring the social data presented since the first version, in which in this second review we updated indicators and the introduction of the issue of Brazilian federalism as presented throughout the manuscript.

21- Are there no differences between regions and cities? How can a “poor” Brazilian family take care of a dependent elderly person? How do different family arrangements manage to care for the elderly? As it stands, there is a homogeneity in the way of thinking about such families' responsibilities and viability. 22- Managers point out in a bureaucratic way, but the analysis needs to counter argue the findings.

R. Based on the previous suggestions, we hope we have answered this question.

**Final considerations**

Insufficient and generic. It needs to answer the research question supported by the results. But bring perspectives of intervention in politics in a less generic way.

We thank you for the suggestion of redesigning the final considerations to remedy the generic and we present this request in the base text.

**Reviewer 2**
The text is confusing sometimes due to the use of prepositions that may not be the most appropriate, literal translation of expressions or idioms that may not have the same meaning in English, and other aspects that require the support of a professional reviewer that may have access to the text written in the Portuguese for the correct alterations.

R. We develop the spelling review of the material with a specialized professional as suggested by the reviewer 2.

As for the structure of the text, in the results section we advise the authors to check how the statements presented may reinforce or build upon what has been presented and will be presented in the discussion section.

R. We verified the empirical material, seeking to reinforce the analysis and discussion, presented throughout the sections as requested.

At one point the authors suggest a comparison between Brazil's health system and other countries, but forget to explain that Brazilian's federalism, consisted of 3 federative beings, is not easily comparable due to its particularities. This makes the implementation of primary health a particular challenge in Brazil. There is a lack of connection of primary care for dependent older adults and the Brazilian family health strategy, this could be an important aspect of the implementation of services.

R. We complement the review questioning, which is presented as follows: Stands out n the Brazilian reality, federalism, composed of three federative entities, is not easily comparable due to its particularities, making the implementation of PHC a particular challenge in Brazil. The disarticulation of PHC and the family health strategy in the care of the dependent elderly suffers from barriers to the implementation of primary services in a universal way.

In discussion, the authors present a figure related to older adults who are responsible for families (line 400), but present an outdated figure, the same author has much recent work on the subject, even including the effect of the Covid-19 outbreak on families and the income of older populations.

R. We have made the necessary update in the text as well as at the end (references): A paradox is currently experienced by families with older adults in their homes. More than 90,1% of these people help maintain or maintain families alone.

Lines 419-421 repeat the figures in the introduction, which are not from the "Censo" but from another household research, make sure you cite the updated values that increase 60+ participation in our society.

R. We chose to promote these data only in the introductory framework, improving the discursive synthesis here.

Content between lines 441-443 were not very clear, and need careful review. Line 477 cites a study by Brazilian authors that is not presented in the reference list.

R. Adjustment made, based on the support material presented. It was without the proper reference.

The last paragraphs before conclusions cite Japanese examples and possible adaptations but does not suggest how to adapt and which services could be formulated for dependent older adults.

R. Reformulation promptly complied with, with base text presented: An example that could be adapted to the Brazilian reality, based on the implementation of long-term care, institutional investments that meet the needs of health managers for the proper consolidation of PHC and the family health strategy. Thus, strengthening intersectoral relationships centered on home care. Since it is necessary to take better care of older adults in Brazil and the world that is aging36-37.

There could be a paragraph where some of the limitations of the study are presented (Why 4 participants in a middle-size city and just 1 in most of the largest cities? How can this short list of professionals limit or influence the results? How was the interview carried and what were some of the possible limitations in the interview script? and other things that they may consider relevant).

R. We have inserted this important paragraph as presented: The limitations of the study include the unavailability of carrying out focus groups between managers and federative units. Another difficulty is highlighted by access to data collection, given the time that management has for the interview, limited physical spaces and contexts of urban violence that directly implicate research processes in Brazil.

We hope our suggestion can improve and better connect what has been presented throughout the article with the relevant debate of the subject

R. We greatly appreciate the reviewer's contributions in fostering valuable improvements to the subject and theme.
